# A multicriteria decision analysis framework for developing and evaluating coastal retreat policy

**DOI:** 10.1002/ieam.4662

**Published:** 2022-09-08

**Authors:** Tyler A. Skidmore, Jared L. Cohon

**Affiliations:** ^1^ Department of Engineering and Public Policy Carnegie Mellon University Pittsburgh Pennsylvania USA

**Keywords:** Climate adaptation, Coastal retreat, Managed retreat, MCDA, Multiple‐criteria decision analysis

## Abstract

Managed retreat may be a necessity for coastal communities as sea levels rise due to climate change. Selecting the right policy decisions and timing is difficult given the vested interests of communities and stakeholder groups and requires careful balancing of the benefits and risks associated with each management alternative. State and federal agencies often employ single‐objective optimization frameworks such as cost‐benefit analysis to analyze coastal relocation alternatives, but such methods are limited in their ability to balance competing value considerations and stakeholder demands. The use of a multicriteria decision analysis (MCDA) methodology allows for such considerations to be quantified and evaluated, thereby improving planning and decision‐making for coastal retreat policies. This paper provides a strategic MCDA framework to evaluate coastal retreat policy that could be leveraged by at‐risk coastal communities. The MCDA is applied to a hypothetical coastal retreat scenario to visualize policy preferences and differing value considerations among stakeholders. This model can be used by government agencies to foster more sound, acceptable, and implementable coastal retreat policies and streamline the incorporation of this climate adaptation mechanism, which may be necessary for the near future. *Integr Environ Assess Manag* 2023;19:83–98. © 2022 The Authors. *Integrated Environmental Assessment and Management* published by Wiley Periodicals LLC on behalf of Society of Environmental Toxicology & Chemistry (SETAC).

## KEY POINTS

This paper presents a multicriteria decision analysis tool that can assist in the development and evaluation of coastal retreat policy and decision‐making, and demonstrates the tool's functionality in a hypothetical coastal retreat scenario.

## INTRODUCTION

Sea level rise and coastal erosion threaten the social, political, and economic well‐being of coastal communities across the United States in a myriad of interconnected ways, and a commitment to adaptation is necessary to mitigate the crises that inaction will cause. The inherent uncertainty as to the precise extent of sea level rise necessitates the development of multiple adaptation pathways and a diverse set of strategies, including but not limited to managed retreat (Haasnoot et al., 2021). Managed coastal retreat is “the purposeful, coordinated movement of people and assets out of harm's way” (Carey, 2020). It involves the planned relocation of communities to areas less at risk from the threats posed by climate change, coastal flooding, or other climate‐related calamities to prevent an inevitable and haphazard evacuation later when individuals decide of their own accord that the area is no longer livable. This method of climate adaptation, when thoroughly planned and consciously executed, can provide numerous benefits to society, such as the reduction of risk, social equity, economic efficiency, and the preservation of community values (Mach & Siders, 2021; Pilkey & Pilkey, 2019, p. 13, p. 14).

Thus far, however, managed retreat has not seriously entered the consciousness of the American body politic as a means of addressing climate change (Teirstein, 2021). Additionally, the United States has not applied a unified approach to climate adaptation or coastal management policy. This may be a necessity to manage a problem like coastal erosion and sea level rise where effects cannot be compartmentalized into the borders of cities, states, or tribal lands, or where adaptation pathway planning may benefit from the common strategy, goal, and purpose among disparate organizations. However, such policies are instead overseen by a patchwork of local, state, and federal government agencies. States have reduced budgets for coastal management programs in recent decades due to coastline deregulation and general skepticism about or denial of climate change (Pilkey et al., 2017). Some local governments have implemented regulations aimed at disincentivizing coastal development, but the existence of the National Flood Insurance Program, which is administered at the federal level by the Federal Emergency Management Agency, makes some of these regulations moot by arguably incentivizing investment on the part of both individuals and corporations into risky shorefront properties by subsidizing damages in the case of a natural disaster (Pilkey & Pilkey, 2019). In short, there is no unified coastal management strategy from which to begin developing a coherent managed retreat plan.

Coastal retreat policy is the subject of much discussion, as many academic circles are increasingly seeing it as a necessary climate change adaptation mechanism (Carey, 2020). However, the decision to retreat must be informed by a wide range of value considerations, including easily quantifiable factors like monetary costs and the preservation of property value, as well as less quantifiable considerations, such as the preservation of culture, community acceptance, and equity, which makes developing these policies difficult on the part of public agencies (Mach & Siders, 2021). The multitude of disparate and potentially conflicting factors requires decisions to be viewed holistically; the most monetarily cost‐effective retreat options may not maximize community values and vice versa. Additionally, different stakeholders who may be affected by the decision to retreat may have different sets of priorities and values, underscoring the need for a policy evaluation framework that can not only synthesize and compare heterogenous policy considerations but “weight” these considerations differently based on stakeholder input (Mach & Siders, 2021).

For example, United States Army Corps of Engineers (USACE) has historically been an agency charged with federal‐level coastal management (Pilkey & Pilkey, 2019; Pilkey & Wheeler, 1998) and is one of the government bodies that may one day be responsible for implementing coastal retreat policy. At the request of the indigenous community in Kivalina, Alaska, USACE conducted an in‐depth assessment of the financial, political, and technical feasibility of entirely relocating villages (Hayes & URS Corporation, 2006). The Inupiat way of life has long been discussed as a potential casualty of climate change; the gradual erosion of the ecosystem due to rising seas threatens their near‐subsistence lifestyle, which is one of the last in North America (Pilkey & Pilkey, 2019, p. 15). In fact, USACE's work in Kivalina reveals that the organization recognizes that a purely monetary framework does not capture the full scope of the values, which must be optimized with regard to coastal relocation; this project would be cost‐prohibitive if factors such as the preservation of the Inupiat culture and way of life had not been given significant consideration.

In this way, coastal relocation is inherently multiobjective, involving many different value considerations and stakeholder priorities. While it is necessary when considering climate adaptation pathways to incorporate cost‐benefit analysis (CBA), which analyzes public policy and regulation from a monetary perspective (De Ruig et al., 2019), financial assessment alone, as has been government practice thus far, will be of limited use. The complex nature of accessing what a “good” managed retreat policy would be (Carey, 2020, p. 1) requires analytical tools that evaluate multiple criteria and go beyond CBA.

While agencies may recognize the need to incorporate multiple criteria into their coastal planning, they have not yet adopted a methodology to support this approach. Multicriteria decision analysis (MCDA) is a policy analysis methodology specifically created for situations like this; it can be used to evaluate management options against several criteria and a broad range of values represented by the relative importance of the criteria to policymakers and stakeholders (Linkov et al., 2020). Despite this ability to provide a reasoned methodological evaluation of these disparate values, it has so far been unused in relocation policy formulation according to publicly available information (Linkov et al., 2020).

This article will demonstrate an MCDA application to provide a novel strategic decision‐making framework for the formulation and implementation of coastal retreat policy. It will provide a broad baseline set of coastal retreat‐specific criteria and evaluation mechanisms for decisionmakers to evaluate and implement coastal retreat policy from a macrolevel perspective. As will be discussed below, the tool in its current form is broadly scoped, allowing organizations to fine‐tune and calibrate it for their specific needs and situation.

## REVIEW OF MCDA METHODS

### Overview

Multicriteria decision analysis is a family of analytical frameworks that has been used in many areas at the intersection of environmental science and policy. Multicriteria decision analysis balances environmental and economic concerns with more intangible policy considerations and visualizes how different stakeholders may place a different emphasis on specific criteria (Linkov et al., 2020). Regardless of which MCDA method is selected, there is a common process for evaluating problems with this framework:

(1) Identifying criteria and their metrics,
(2) identifying alternatives and their scoring against said criteria, and
(3) incorporating policymaker and/or stakeholder preferences for the relative importance of the criteria, for example, weights, such that the scores from (2) can be combined and evaluated to indicate a preferred alternative.

The major difference among MCDA methods is the specific ways in which preferences are elicited and combined to evaluate alternatives in (3).

Multicriteria decision analysis has been used with similar focus topics and other coastal planning mechanisms. For example, Trump et al. (2018) demonstrate how MCDA can be used to balance political and economic values with environmental concerns among stakeholders in the Arctic. Roca et al. (2008) utilize a participatory approach and conduct interviews with a wide variety of stakeholders to elicit alternative preferences regarding coastal erosion in Lido De Sete, France. Through this approach, policymakers can shape the public response to coastal erosion in a manner that more closely reflects stakeholder preferences. Lawrence et al. (2019) use MCDA to address uncertainty regarding climate change for the development of a coastal adaptation strategy in Hawke's Bay, New Zealand using stakeholder elicitation, dynamic adaptive pathways planning (DAPP), and real options analysis. In this circumstance, MCDA provides a structured way for stakeholders and decisionmakers to frame and interact with coastal retreat challenges. It is a tool that, in addition to potentially providing the best course of action, helps those involved articulate preferences and increase understanding of the problem and the alternatives.

### Method selection

Widely used MCDA methods include the Analytic Hierarchy Process (AHP), Preference Ranking Organization Method for Enrichment of Evaluations, and Multi‐Attribute Utility Theory (MAUT)‐based methods (Figueira et al., 2005). Each has its own mathematical justifications, as well as camps of supporters and detractors (Forman & Gass, 2001; Velasquez & Hester, 2013). Although no MCDA method has been used for a coastal retreat problem, USACE and other government agencies are aware of and employ MCDA in a variety of other problem settings (Linkov et al., 2020, p. 20, p. 21). Multi‐Attribute Utility Theory is a frequently used MCDA method by government agencies, followed by AHP (Linkov et al., 2020, p. 28). For this reason, a MAUT‐based methodology was selected for this coastal relocation framework.

Each MCDA method has its own limitations: for example, MAUT is “incredibly data‐intensive,” requiring a level of decisionmaker input and preference specificity, which may not be feasible for every decision problem (Velasquez & Hester, 2013). The AHP allows stakeholders to weigh criteria and compare alternatives with ease that MAUT does not afford, but the method is susceptible to rank reversal such that “problems where alternatives are commonly added would do well to avoid this method” (Velasquez & Hester, 2013). Thus, a simplified version of MAUT that greatly reduces the data intensity and demands on policymakers and stakeholders was selected.

## METHODOLOGY

This model uses the Simple Multi‐Attribute Rating Technique (SMART). As a version of MAUT (Keeney & Raiffa, 1993), this method requires:

(1) Establishing or assuming mutual preferential independence (MPI), a condition where criteria preferences are independent of each other.
(2) Determining the value function, which is assumed to be additive due to MPI, as well as linear in the case of SMART.
(3) Applying the value function to find the best alternative.

The consequence of the additivity and linearity assumptions is that the value function consists of weights on the criteria and the overall score of each alternative is just the sum of the individual criteria scores multiplied by their respective weights.

$$U(i)=\sum_{k}(\lambda_{k})z_{ik},$$

where U(i) is the overall score of alternative i, $z_{\mathrm{ik}}$ is the score of alternative i on criterion k, and $\lambda_{k}$ is the weight on criterion k.

This method was selected because it is relatively easy for decisionmakers to understand and use, and is not that different from MCDA methodologies already employed by government agencies. This maximizes its versatility for government agencies, which may not be formally trained in the use of MCDA, and allows for more candid stakeholder input from individuals and groups with limited experience with this methodology as well. Additionally, it addresses the weaknesses of pure MAUT, such as its data intensiveness and difficulty of use. However, these advantages are bought at the price of simplifying assumptions about preferences. These assumptions could be changed, or other MCDA techniques could be applied, but the use of SMART, despite its simplified assumptions, provides an effective way to foster stakeholder discussions about values and incorporate them into the analysis. This offers a major step forward for governmental coastal management.

### Selection of areas to inform criteria selection

Climate change and coastal erosion affect coastal communities in different ways. To develop a broad framework that is applicable to the dozens of areas that may need to consider coastal retreat, this study selected specific areas to frame the underlying assumptions and criteria needed to characterize the model. Localities were selected based on a preexisting decision to pursue a coastal retreat policy in the area or the likelihood of a need to execute a managed retreat in that area in the future. The areas selected for consideration and their existing USACE Coastal Systems Portfolio Initiative Projects can be found in Table 1.

**Table 1:** USACE Coastal Systems Portfolio Initiative (CSPI) Projects

Study site	CSPI Project names
Kivalina	Kivalina Coastal Erosion
Shishmaref	Shishmaref Coastal Erosion
Martha's Vineyard	Vineyardhaven Harbor
	Lagoon Pond
	Oak Bluffs Harbor
	Edgartown Harbor
	Menemsha Creek
Miami	Dade County BEC—Sunny Isles
	Bakers Haulover Inlet
	Miami‐Dade Back Bay CSRM
	Dade County BEC—Main Segment
	Intracoastal Waterway—Jacksonville to Miami (IWW)
	Government Cut/Miami Harbor
New Orleans	Lake Pontchartrain and Vicinity
	Southeast Louisiana Urban Flood Damage Reduction Project (SELA)
	GIWW: Harvey Lock Forebay
	GIWW: IHNC Lock Forebay
	GIWW: Algiers Lock Forebay

The decision was made to examine a set of sites because the unique priorities, existing coastal policies, demographic and social structures, and coastal projects of a set of areas allow one to discern commonalities among localities that may need to consider coastal retreat. This helps to frame the MCDA tool such that it is broad enough to be useful in a multitude of areas and further the goal of a unified coastal management strategy. However, this helps the tool become specific enough to be relevant to a multitude of coastal retreat scenarios. Care was taken to study a diverse set of areas to ensure that a wide set of potential stakeholder needs would be represented among the criteria. Thus, the selected areas discussed below include relatively affluent areas, rural and tribal localities, and large cities, which are politically, culturally, and socioeconomically heterogenous.

#### Kivalina

Kivalina is a native Inupiat community of roughly 400 people in the far reaches of Alaska that is already experiencing the effects of climate change‐induced coastal erosion (Hamilton et al., 2016; US Census Bureau, 2022). As stated before, the ecosystem upon which the community depends for their subsistence culture is rapidly disappearing (Pilkey & Pilkey, 2019). Thinning sea ice and melting permafrost have altered local hunting patterns (Pilkey & Pilkey, 2019). These issues are compounded by the fact that the people of Kivalina cannot simply move to a city; among other problems, they have few skills that would be marketable in today's economy (Pilkey & Pilkey, 2019). USACE is currently heavily involved in the area; they built a seawall as a temporary measure (Pilkey & Pilkey, 2019, p. 21), and conducted studies and produced an in‐depth technical report outlining stakeholders, costs, and values (Hayes & URS Corporation, 2006). This report, as well as the unique struggles of the Kivalina community, greatly informed the scope of this MCDA tool.

#### Shishmaref

Like Kivalina, the locality of Shishmaref is also facing the gradual erosion of its traditional ecosystem, which is worrisome to the Inupiat hunter‐gatherers. Shishmaref lies north of the Bering Strait, and the area has been described as having the most pronounced climate change‐related damage in the world (Willis, 2004). USACE is also involved in the area and has made public its plans to ultimately relocate the village, but existing public relocation plans are currently not as developed as those of Kivalina (USACE Alaska District, 2022).

#### Martha's Vineyard

Martha's Vineyard is an island off the coast of Massachusetts. The permanent population of the area is about 17 000, but as a common summer destination for relatively wealthy people, the population can reach about 100 000 during the summer months (US Census Bureau, 2021). This locality was selected because of the set of ongoing coastal erosion‐related issues that the municipal government is attempting to manage. Unlike Kivalina and Shishmaref, it is relatively affluent by traditional metrics. The Martha's Vineyard Commission, the local governmental agency in charge of land management, is currently making plans for implementing some form of coastal retreat policy (Martha's Vineyard Commission, 2022).

#### Miami

The future of Miami, Florida and its outlying areas is often invoked when discussing the necessity for a coastal retreat policy. Miami is a large city of about 440 000 with a massive metropolitan area of about 6 million (US Census Bureau, 2010). Miami sits upon a bedrock of porous limestone, which will exacerbate the effects of sea level rise in the area, as flooding will become more and more common. This makes considering retreat of the area a necessity (YCC Team, 2020). Miami already experiences many of the impacts generally attributed to sea level rise, including periodic nuisance flooding (Pilkey & Pilkey, 2019). USACE has historically been involved in many hard stabilization projects in the vicinity, but as stated before, these solutions are only temporary, and a long‐term solution to flooding will soon be necessary (Coastal Systems Portfolio Initiative, 2022).

#### New Orleans

New Orleans is also one of the most frequently cited cities when it comes to visualizing the effects of climate change. A city of about 400 000 with an outlying area of 1.5 million, it is demographically and culturally diverse (The Data Center, 2021). Its economy is heavily dependent on tourism and, like Miami, New Orleans and the surrounding area exist in a precarious relationship with the wetlands, upon which the city is built, and which surrounds it (Jacobson, 2012). USACE is currently involved in a set of projects for New Orleans and the surrounding areas, but, as with Miami, no large‐scale coastal retreat policy has been proposed (Coastal Systems Portfolio Initiative, 2022).

### Criteria identification and selection

**Figure 1:**
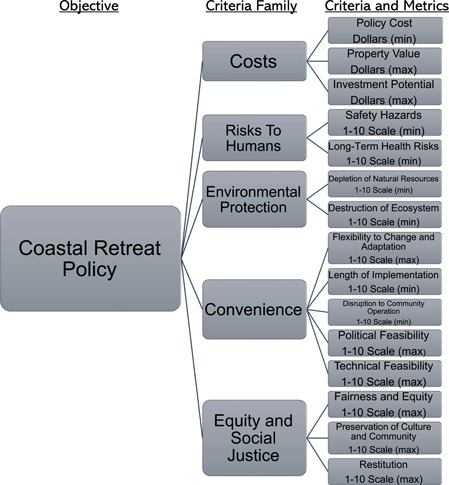
Criteria hierarchy of the MCDA model. The fundamental objective, criteria families, and individual criteria are the top, middle, and bottom levels, respectively. MCDA, multicriteria decision analysis

These case studies, as well as broader coastal retreat policy research, helped to identify the criteria that could capture the unique needs of these localities (Carey, 2020; Mach & Siders, 2021; Pilkey & Pilkey, 2019). Additionally, input from personnel in USACE Engineering Research and Development Center (ERDC), a component of USACE tasked with forward‐looking environmental science research for use by the organization, informed the structure of this MCDA tool (I. Linkov, USACE Engineer Research and Development Center, personal communication, April 4, 2022). To further assist in framing this problem, meetings with the Martha's Vineyard Commission in Massachusetts, the municipal government, and the ERDC were conducted to gain a sense of stakeholder perspectives on which criteria ought to inform policy analysis (Martha's Vineyard Commission, personal communication, April 15, 2022). Their contributions will be outlined in the “Model” section. With these inputs, criteria and metrics were identified and organized in a criteria hierarchy, pictured in Figure 1, which shows criteria “families,” that is, groupings of criteria and related subcriteria. Metrics for evaluating alternatives to these criteria are also shown.

## MODEL

One of the initial issues in framing this problem is that many of these criteria could potentially be endlessly disaggregated. As a result, many criteria were considered but then discarded in the interest of keeping the model as broad as possible to be useful to a multitude of scenarios, but sufficiently specific to analyze individual cases. Criteria that overlap could also potentially violate MPI, as they would not be entirely independent of one another. Finally, a balance had to be struck so that there were enough criteria to capture the full scope of the problem, without having so many criteria that stakeholders and policymakers would be overwhelmed. This is a common challenge in applying MCDA; see, for example, Hammond et al. (2002). As it is, there are 15 criteria, a large number but not unusual for problems of this complexity. Grouping related criteria into five criteria families helps users deal with so many factors.

Additionally, discussions with USACE personnel revealed a desire to make the model useful for stakeholder engagement (I. Linkov, USACE Engineer Research and Development Center, personal communication, 2022). In this sense, the model serves two purposes. It is both a framework that agencies can show to stakeholders and communities to gauge their preferences, and a framework to be used internally to structure, evaluate, and implement coastal retreat policy.

Table 2 lists all criteria and provides a brief description of each. Note that, other than the cost criteria, all the criteria are measured on a 1–10 interval scale. This reflects the inherently subjective nature of many of these criteria, for example, preservation of culture and community. The criteria in the Risk and Environmental Protection categories could be measured with ratio scales, or surrogate measures, but that would require specification of additional subcriteria adding complexity for stakeholders and diminishing the overall value of the other criteria. Each criterion is discussed further in the following sections.

**Table 2:** Criteria families

Criteria family	Subcriteria	Metrics	Description
Costs	Policy cost	Dollars (min)	The total cost taken on by government agencies because of a policy, or costs borne by government agencies due to inaction.
	Property value	Dollars (max)	The impact of a specific policy on the property value of a given area.
	Investment potential	Dollars (max)	The monetary potential for private or public investment into an area because of a specific policy.
Risks to humans	Safety hazards	1–10 Scale (min)	The risk of immediate danger or death because of a specific policy (falling rocks, construction hazards, etc.)
	Long‐term health risks	1–10 Scale (min)	The long‐term health risks of moving to a specific area, including the effects of pollution on individuals.
Environmental protection	Depletion of natural resources	1–10 Scale (min)	The effect on the depletion of natural resources, including fossil fuels, minerals, and others, in a specific area.
	Destruction of ecosystem	1–10 Scale (min)	The effect on the degradation or damage to a natural ecosystem or habitat.
Convenience	Flexibility to change and adaptation	1–10 Scale (max)	The ability of a policy to be changed after its initial selection.
	Length of implementation	1–10 Scale (min)	The length of time a policy would take to implement from start to completion.
	Disruption to community operation	1–10 Scale (min)	The impact of how disruptive a policy is to a community's commerce, daily operation, quality of life, etc.
	Political feasibility	1–10 Scale (max)	The level of acceptance or resistance to a specific policy from a community or other government agency that may frustrate policy implementation.
	Technical feasibility	1–10 Scale (max)	The ability for the policy to be technologically implemented, including geological suitability, technological limitations, etc.
Equity and social justice	Fairness and equity	1–10 Scale (max)	The fairness across socioeconomic, cultural, ethnic, or racial lines of a specific policy, which includes environmental and distributive justice.
	Preservation of culture and community	1–10 Scale (max)	The effect of a specific policy on the preservation of a specific culture, way of life, or community.
	Restitution	1–10 Scale (max)	The ability of a specific policy to potentially address a historical inequity.

### Criteria and criteria families

#### Costs

The criteria family “Costs” includes monetary considerations of coastal retreat policy. These considerations would be important to a myriad of stakeholders, including government agencies, taxpayers, and more, whose support may be critical to enacting coastal retreat policy. Each of these criteria could be measured with a net present value over a specific time horizon (Morgan, 2017).

1. *Policy cost*: This criterion measures the amount of money that a policy alternative will cost. It is reasonable for the public to desire their government to undertake policies that do not result in significant public debts for little payoff, or which increase taxes beyond that which is reasonable. Aspects of coastal management policy have been criticized for their lack of care in this domain in the past (Carey, 2020). Moreover, agencies responsible for devising and implementing coastal retreat policies have limited budgets, and certain policies may prove to be inadvisable if monetarily infeasible (Hamilton et al., 2016; Hayes & URS Corporation, 2006).
2. *Property value*: This measures how an alternative preserves property value, which is relevant for any coastal retreat policy, especially in areas such as Miami where this has been a dominant consideration in existing coastal management policy (Anderson, 2022; Carey, 2020; Pilkey & Pilkey, 2019). Property owners, whose support may be necessary to implement such policies, would likely be very concerned about this criterion, but in many cases, this criterion may not be applicable or must be applied along a specified time horizon, especially in areas where the property will not exist indefinitely.
3. *Investment potential*: This criterion measures the likelihood of ongoing public and private monetary investment in an area. This is a concern for areas like Miami, New Orleans, and Martha's Vineyard, where a decline in commercial or public investment could result in significant degradation of the quality of life for the residents (Pilkey & Pilkey, 2019; Pilkey et al., 2017). This criterion is intentionally framed broadly to account for a variety of situations.

#### Risks to humans

The “Risks To Humans” criteria family considers risks to the health and safety of both the community that will be affected by retreat policy or inaction and those tasked with implementing it.

4. *Safety hazards*: This criterion measures immediate risks of death or injury resulting from a specific policy or inaction. Government agencies have expressed concerns regarding the safety of implementing specific coastal retreat policies (Hayes & URS Corporation, 2006).
5. *Long‐term health risks*: This criterion measures the long‐term health impacts of a specific policy or inaction. For example, a policy may create conditions where individuals or communities are exposed to pollution, or a policy can lead to sewage and sanitation infrastructure issues, both of which would create the risk for disease spread and other long‐term health issues (Hayes & URS Corporation, 2006).

#### Environmental protection

This criteria family includes considerations for the protection and preservation of the environment. A broad array of stakeholders may care about this criteria family, including the general public, conservationists, activists, and more. These impacts must be considered in any action taken by a government agency.

6. *Depletion of natural resources*: This criterion considers the use or destruction of natural resources because of a specific policy or inaction, including natural gas and fossil fuels, logging, mining, hunting, and others.
7. *Destruction of ecosystem*: This criterion measures the level of environmental damage, which will be caused by a policy or inaction. Discussions with the Martha's Vineyard Commission revealed a desire to forestall damage to the area's marshland, which supports much of the life on the island. Moreover, coastal adaptation techniques, which employ hard stabilization, such as the construction of seawalls and beach replenishment, gradually erode the ecosystem of the beach and may reduce biodiversity (Gittman et al., 2016; Pilkey & Wheeler, 1998). Policies taken on by the government must seek to mitigate such damage, and this must be accounted for in policy analysis.

#### Convenience

The criteria family measures considerations of a policy or inaction that will result in technical, political, or other problems, which may require sustained stakeholder or agency engagement. Essentially, this criteria family is meant to measure the subjective amount of “trouble” a policy takes to implement, or which is caused by inaction.

8. *Flexibility to change and adaptation*: This criterion subjectively measures how permissive a specific policy is to change over time *after* it has already been selected, and how much time this could potentially take. Technologies improve, costs change, community values shift, and political situations evolve with time. Coastal retreat policies may need to change considering these developments (Mach & Siders, 2021, p. 2).
9. *Length of implementation*: This criterion measures how quickly a policy can be implemented from start to finish. In theory, a quicker policy to implement would be more favorable than one which takes longer. The Martha's Vineyard Commission expressed the desire for this to be considered.
10. *Disruption to community operation*: This measures the disruption a policy or inaction may impose on a particular community's activities—social, cultural, and economic—while the policy is being implemented and afterward. The disruption to existing institutions in particular communities will reduce long‐term community health, so a policy meant to assist communities ought to consider this aspect of coastal retreat policy (Carey, 2020; Mach & Siders, 2021).
11. *Political feasibility*: This criterion measures the level of bureaucratic or community acceptance or resistance to a specific policy or inaction. A specific policy may be met with resistance from other federal agencies, or state and local governments (Teirstein, 2021). Additionally, existing public agencies may not be empowered to address the specific and niche needs of communities, as can often be the case with Native Alaskan areas (Shearer, 2012). Moreover, communities may simply not want to move, an especially pressing concern in Kivalina and Shishmaref (Hayes & URS Corporation, 2006). Given the fraught history of “relocation” of indigenous peoples, this aversion should not be a surprise. Policies should seek as much community support and input as possible.
12. *Technical feasibility*: This criterion seeks to measure the feasibility of an alternative from an engineering perspective. This includes considerations of the geological suitability of an area, the feasibility of infrastructure development, the usability of coastal transportation infrastructure to conduct the retreat, the area's ability to sustain population growth, and more. These concerns especially affected certain alternatives in Kivalina (Hamilton et al., 2016), but this criterion is intentionally broadly scoped to allow the evaluation of a multitude of areas with differing engineering concerns.

#### Equity and social justice

This criteria family includes considerations regarding distributional impacts and other social, political, and economic equity issues. Having historically been neglected in a wide array of public policy fields, equity is likely to be an issue in most coastal retreat projects, which can either exacerbate inequity or be used to advance societal values (Carey, 2020; Mach & Siders, 2021). Activist groups and native communities whose support, “buy‐in,” and input may be critical to implementing the sound policy would certainly care about these considerations.

13. *Fairness and equity*: This criterion examines the distributional impacts of a specific policy or inaction, which includes environmental justice. For example, less economically advantaged communities may be in areas where the effects of inaction will be felt most acutely, such as in New Orleans (Mach & Siders, 2021; Pilkey & Pilkey, 2019). In another example, stakeholders or policymakers may not find it fair to ask certain populations to move, an especially pressing concern regarding native communities and the fraught history of “relocation.” Finally, the actual implementation of a specific policy may be conducted in a manner with harmful distributional impacts (Siders, 2019). This criterion is intentionally scoped broadly so that it can be applied to a multitude of areas.
14. *Preservation of culture and community*: A new policy or inaction may set conditions where a specific culture, community, or way of life is less likely to be protected, an especially important consideration with indigenous communities (Mach & Siders, 2021). For example, the sense of “place” is such a crucial aspect of the Inupiat culture that moving is virtually unthinkable (Agyeman et al., 2009). A hypothetical example would be a policy decision to integrate Inupiat people into Alaskan cities, thereby eroding their unique lifestyle (Pilkey & Pilkey, 2019).
15. *Restitution*: This criterion seeks to measure the extent to which a new policy or inaction may serve to redress a historical inequity, or to “right” a historical wrong. Managed retreat policy, when planned and executed effectively, can be used in furtherance of broader societal interests, including addressing historical inequities (Mach & Siders, 2021). When assessing coastal retreat policy for minority and indigenous communities, agencies must consider if their policies could potentially advance the objectives of broader social equality.

### Alternatives

Alternatives are the potential choices that a model evaluates based on their performance according to the criteria. For this model, the alternatives would be potential coastal retreat policies or plans, which are developed by relevant decisionmakers. A “base case” or “do nothing” alternative should be included in every situation. This alternative must be evaluated in the same manner as other coastal retreat alternatives, which is discussed in the next section. For example, in USACE Kivalina Report, USACE outlines specific potential policy choices, such as “do nothing,” “improve the current site,” or “move the village to a new site” and provides cost estimates for each (Hayes & URS Corporation, 2006).

While Kivalina is used here as an example, this model is meant to be applicable to any locality where the coastal retreat is being considered. As such, the model in its current form does not “come with” preprescribed alternatives, nor does it advise decisionmakers on how to define alternatives; they will need to be supplied by decisionmakers based on the specific situation of a particular locality and the decisionmaker's professional judgment.

### Criteria scoring

Each alternative is evaluated in terms of each criterion. For example, the subcriterion “Policy Cost” is evaluated using dollars as a metric, but the subcriterion “Restitution” is evaluated using a 1–10 scale, where 1 is the “worst” and 10 is the “best.” The scores on each criterion, irrespective of metric, are then normalized on a scale from 0 to 1, with 0 as the “worst” and 1 as the “best” (Keeney & Raiffa, 1993). This way, unlike terms such as monetary costs and performance measures on interval scales of 1–10 can easily be compared against each other.

Most criteria are evaluated using a 1–10 interval scale. The qualitative nature of criteria like “Political Feasibility” makes them unavoidably subjective, requiring human intuition for their evaluation. This is not unusual in the field of MCDA; Trump et al. (2018) use a 1–10 scale to evaluate qualitative criteria like the “social” impacts of Arctic policy alternatives. Because of this, however, the onus is on the policymakers and decisionmakers using this model to be alert for and minimize bias. Since the organization which will be scoring alternatives may also be the same organization implementing coastal retreat policy, there is the potential for organizational interests to influence subjective judgments. This is a problem that may be unavoidable when using MCDA (Roy, 2005), but policymakers will need to manage the potential for bias to achieve a meaningful result.

### Stakeholders and criteria weighting

Criteria weights capture how much a decision‐maker or stakeholder “cares” about a specific criterion relative to the others. The weights represent the principal way in which values are expressed and affect policy outcomes. As values vary among stakeholders, we should expect weights to differ among participants in policymaking as well. This is likely especially the case for coastal retreat policy, as there are many different stakeholders who may place different levels of importance on the criteria (Mach & Siders, 2021). USACE, the military, more broadly, state governments, local governments, business owners, property owners, community members generally, and activists will undoubtedly have different evaluations of coastal retreat criteria. Additionally, stakeholders may not have strong revealed preferences regarding the coastal retreat, and they may have little expertize or experience with these issues or in articulating their values. As noted by Velasquez and Hester (2013) it may prove difficult for many stakeholders to select weights. Asking them to do so assumes that either stakeholders have clear and defined preferences, which may be an unreasonable expectation, or that government agencies will be able to draw this information from stakeholders, which could prove to be a time‐consuming and exhausting exercise. It is for these reasons that we made value elicitation especially easy for stakeholders, allowing them to rank‐order criteria preferences from most to least important. These ranks are converted to weights using the corresponding values in Table 3. For example, with a set of three criteria, if a decisionmaker ranks a criterion as 1, meaning the most important, it would receive a weight of 0.5. The criteria ranked as 2 would receive a weight of 0.33, and the third and least important would receive a weight of 0.17.

**Table 3:** Criteria ranks to weights

Number of criteria	9		8		7		6		5		4		3		2	
Weight	1	0.2	1	0.22	1	0.25	1	0.29	1	0.33	1	0.4	1	0.5	1	0.67
	2	0.18	2	0.19	2	0.21	2	0.24	2	0.27	2	0.3	2	0.33	2	0.33
	3	0.16	3	0.17	3	0.18	3	0.19	3	0.2	3	0.2	3	0.17		
	4	0.13	4	0.14	4	0.14	4	0.14	4	0.13	4	0.1				
	5	0.11	5	0.11	5	0.11	5	0.1	5	0.07						
	6	0.09	6	0.08	6	0.07	6	0.05								
	7	0.07	7	0.06	7	0.04										
	8	0.04	8	0.03												
	9	0.02														

Converting ranks to numerical weights is a significant assumption as it creates specificity and concreteness, which may not be justified or accurate. It will be important to conduct an extensive sensitivity analysis on the weights to understand the importance of this assumption. Though the decision was made to rank criteria here, this methodological step can easily be changed.

## ILLUSTRATIVE CASE STUDY

### Decision context and usage

The intent of this model is to provide policymakers with a decision aid. What follows is a narrative explaining how this model would be used in context. This is a model for use when the decision to relocate has either already been made or is being deeply considered beyond conjecture.

Strategic‐level leadership must empower local, geographically oriented leaders to leverage and tailor this model for the specific needs of their geographic command. This model is developed so that government agencies and those in their charge can use it to study the needs of the communities they are responsible for. These agencies must conduct in‐depth fieldwork and research, including liaising with local communities, to develop feasible alternatives for the coastal retreat in each area, based on the specific requirements, situations, and needs of that specific locality. These needs will be different in each area, which is why this model must be used in a decentralized, case‐based manner. Alternatives should always include a base case, or “do nothing” alternative. USACE Kivalina Report (Hayes & URS Corporation, 2006) provides an example of the level of depth this alternative analysis requires.

Evaluating alternatives in terms of the criteria is also a data‐intensive process, requiring engineering and economic analysis, stakeholder interviews, and more. The use of the 1–10 scales to subjectively access and score the “softer” criteria will require human intuition, but it must be informed by in‐depth research, potentially including expert elicitation (Morgan, 2017) on how the alternatives would impact the community. As noted before, there is the temptation for a self‐serving bias at this stage, and policymakers must hold themselves to the highest standards of professionalism to mitigate this.

Agencies should then develop stakeholder profiles based on interviews and elicitations with stakeholder groups to determine how they would rank the specific criteria. Then, policymakers input these data into a model, determine preferred outcomes based on those ranks, and work to find common ground in areas where stakeholders may differ. As stated before, the utility of having stakeholders *rank* preferred criteria is that this method can be applied to the public, without the need for extremely well‐defined preferences.

**Figure 2:**
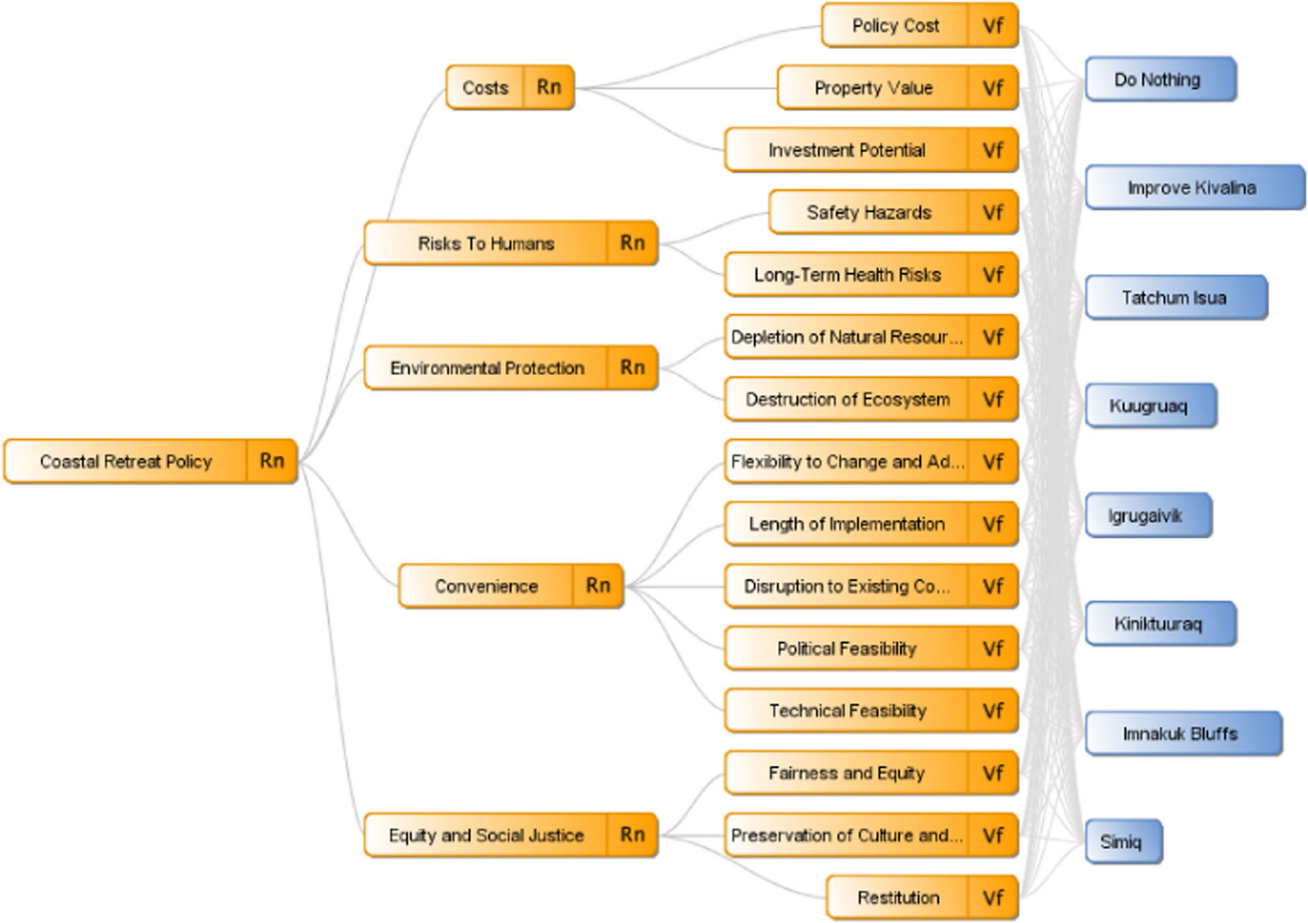
A visual representation of the criteria hierarchy as a model in Decerns, a program built for MCDA. All columns are the same as in Figure 1, except for the new bottom row, which are the potential alternatives for the illustrative case study. MCDA, multicriteria decision analysis

This process can be iterative and does not necessarily need to be completed in this order; if new information is discovered at one phase of this process that may influence another, there is no need to follow a rigid step‐by‐step process. As stated before, part of the utility of MCDA is not merely that it is a decision aid, but also that it can help foster deliberate thought about this problem and generate increased engagement and understanding among policymakers, stakeholders, and the public at large (Figure 2).

### Notional scenario

**Figure 3:**
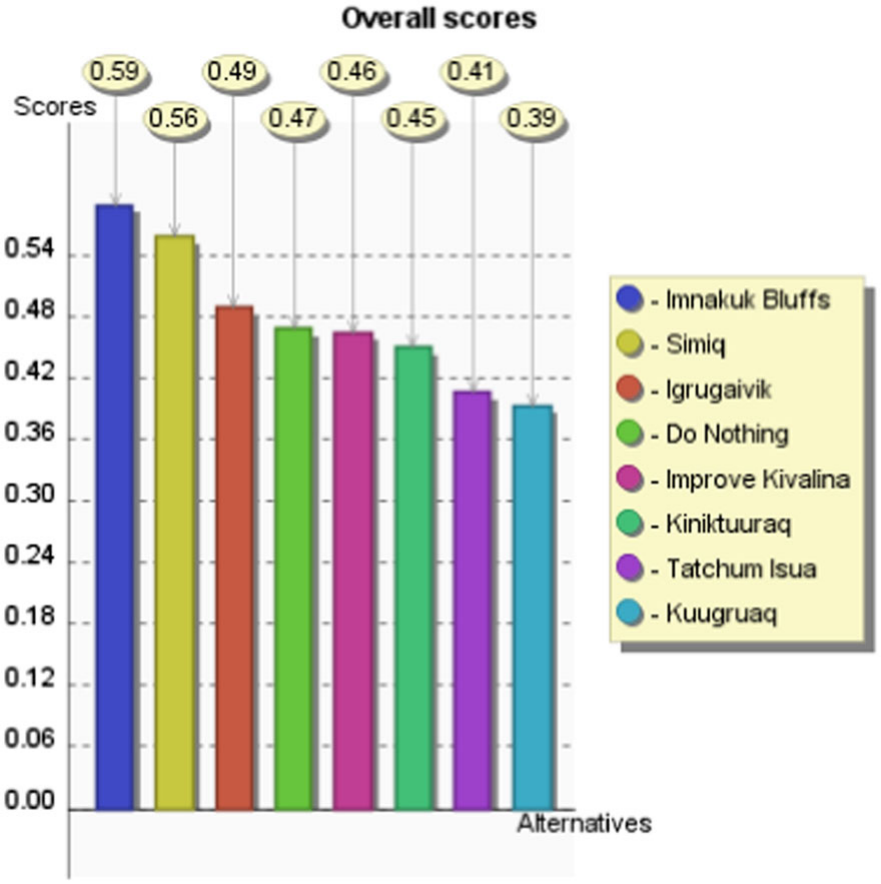
The output of the model for Stakeholder 1's preferences. Stakeholder 1's top alternatives are Imnakuk Bluffs, Simiq, and Igrugaivik. Since Imnakuk Bluffs only barely outperforms Simiq, a decisionmaker should further analyze both alternatives before making a final decision

**Figure 4:**
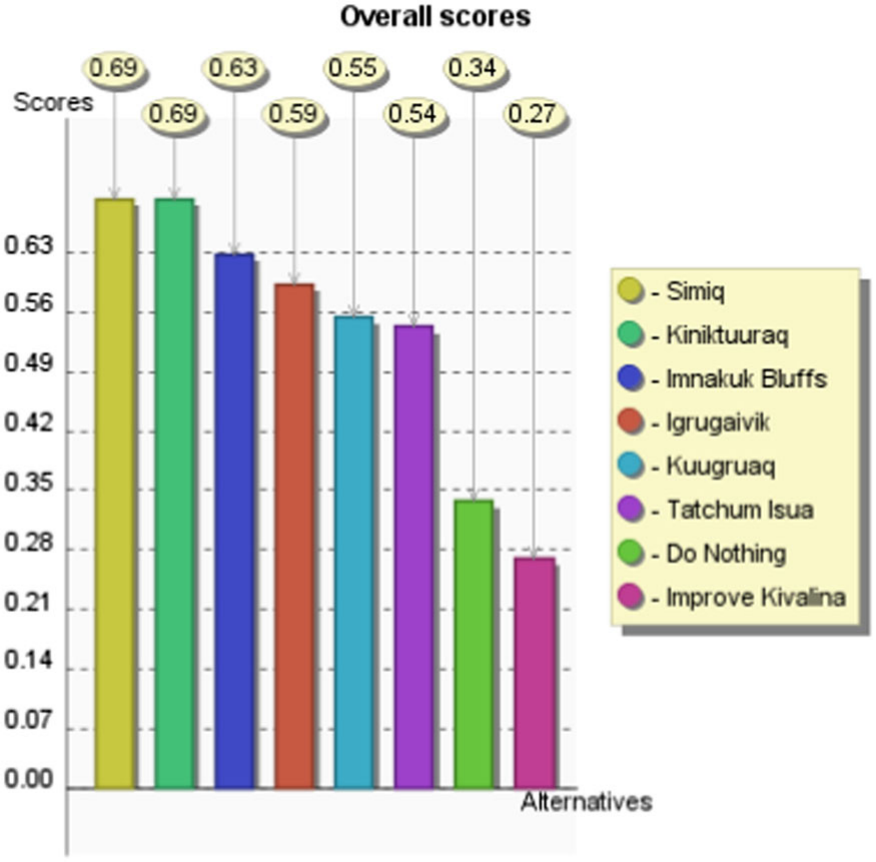
The output of the model for Stakeholder 2's preferences. Stakeholder 2's top alternatives are Simiq, Kiniktuuraq, and Imnakuk Bluffs in that order, indicating potential common interests with Stakeholder 1 that could be mediated

To demonstrate how this model could work in practice, a theoretical decision problem was developed based on the statistics included in USACE Kivalina Report with additional notional information added to evaluate presented alternatives against criteria. For example, the policy costs provided in the report are used, and the contents of the report in a broad sense informed how criteria were scored. This demonstration displays the functionality of this MCDA model by developing and scoring alternatives and applying stakeholder weights to arrive at preferred policy alternatives. A complete listing of notional alternatives, criteria scores, and hypothetical stakeholders is presented in Table 4. Stakeholder 1 is meant to represent a hypothetical landowner, whereas Stakeholder 2 is meant to represent a hypothetical environmental activist. The results of the model, as well as the sensitivity analysis, are pictured in Figures 3 and 4 as well as Table 4, to be discussed in the following section.

**Table 4:** Notional data table

	Criteria						
	Costs			Risks to humans		Environmental protection	
	Policy cost (min)	Property value (max)	Investment potential (max)	Safety hazards (min)	Long‐term health risks (min)	Depletion of natural resources (min)	Destruction of ecosystem (min)
Alternatives							
Do Nothing	$0.00	$200 000	$700 000	9	9	2	6
Improve Kivalina	$196 200 000	$650 000	$1 000 000	7	7	4	8
Tatchim Isua	$154 900 000	$300 000	$900 000	6	5	3	3
Kuugruaq	$245 600 000	$500 000	$600 000	7	3	3	2
Igrugaivik	$246 100 000	$450 000	$1 500 000	5	6	2	2
Kiniktuuraq	$248 200 000	$475 000	$2 000 000	8	7	5	1
Imnakuk Bluffs	$248 700 000	$750 000	$2 500 000	4	3	6	3
Simiq	$251 500 000	$550 000	$1 700 000	4	5	2	1
Stakeholders							
1: Criteria family	1			3		4	
Subcriteria	2	1	3	1	2	1	2
2: Criteria family	5			4		2	
Subcriteria	2	3	1	2	1	2	1

	Criteria							
	Convenience					Equity and social justice		
	Flexibility to change and adaption (max)	Length of implementation (min)	Disruption to community operation (min)	Political feasibility (max)	Technical feasibility (max)	Fairness and equity (max)	Preservation of culture and community (max)	Restitution (max)
Alternatives								
Do Nothing	9	0	1	7	9	0	0	0
Improve Kivalina	6	4	4	8	8	0	3	0
Tatchim Isua	3	5	7	5	7	5	6	4
Kuugruaq	2	8	7	6	6	6	6	3
Igrugaivik	5	6	6	4	7	5	5	5
Kiniktuuraq	6	7	3	9	5	9	7	8
Imnakuk Bluffs	3	6	5	6	6	7	7	6
Simiq	5	6	8	4	7	6	8	6
Stakeholders								
1: Criteria family	2					5		
Subcriteria	2	3	1	4	5	2	1	3
2: Criteria family	3					1		
Subcriteria	5	3	4	1	2	2	3	1

Decerns (Decerns MCDA, 2022), which is software built for MCDA analysis, was used to implement the methodology and apply it to the notional problem.

## RESULTS AND DISCUSSION

### Model outcomes

Based on the notional data found in Table 4 and the preferences of Stakeholder 1, the highest scoring alternative is Imnakuk Bluffs with a score of 0.59, followed closely by Simiq, with a performance score of 0.56, as seen in Figure 3. This visualization is important for decisionmakers so that among other things, agencies using this model can understand the relative distance between preferred alternatives to make better informed decisions, as is one of the goals of MCDA (Roy, 2005). For example, while Imnakuk Bluffs is the preferred alternative, it does not outperform Simiq by a large margin, meaning that these two alternatives may warrant further analysis by decisionmakers before a final policy decision is made.

When one runs this model with the exact same set of alternatives and scoring, but with the weights provided by Stakeholder 2, the preferred alternative is Simiq with a score of 0.69, barely outperforming Kiniktuuraq, and with the Imnakuk Bluffs placing third with 0.63. This is pictured in Figure 4. It appears that while these stakeholders may have different values and ideal choices, both stakeholders rank Imnakuk Bluffs and Simiq in their top three potential alternatives. In this way, visualizing differing stakeholders' weights provides more decision clarity, as it gives the relevant agency important information that could aid in a potential reconciliation between stakeholders' preferred alternatives, in this case, Imnakuk Bluffs and Simiq.

### Sensitivity analysis

Through sensitivity analysis, one can examine in detail how changes in the weights on one or more criteria affect the preferred alternative. In Table 5, one can see how the preferred alternative would change given an increase or decrease in weight for a particular criteria family, with all other weights held constant. In the pictured graphs, each colored line corresponds to the performance of an alternative, whose colors are represented in the key. The *y*‐axis represents the overall performance score of alternatives, where a higher score would imply that an alternative is more preferred. Finally, the *x*‐axis refers to the weight placed on a specific criteria family.

**Table 5:** Sensitivity analyses of criteria family weights

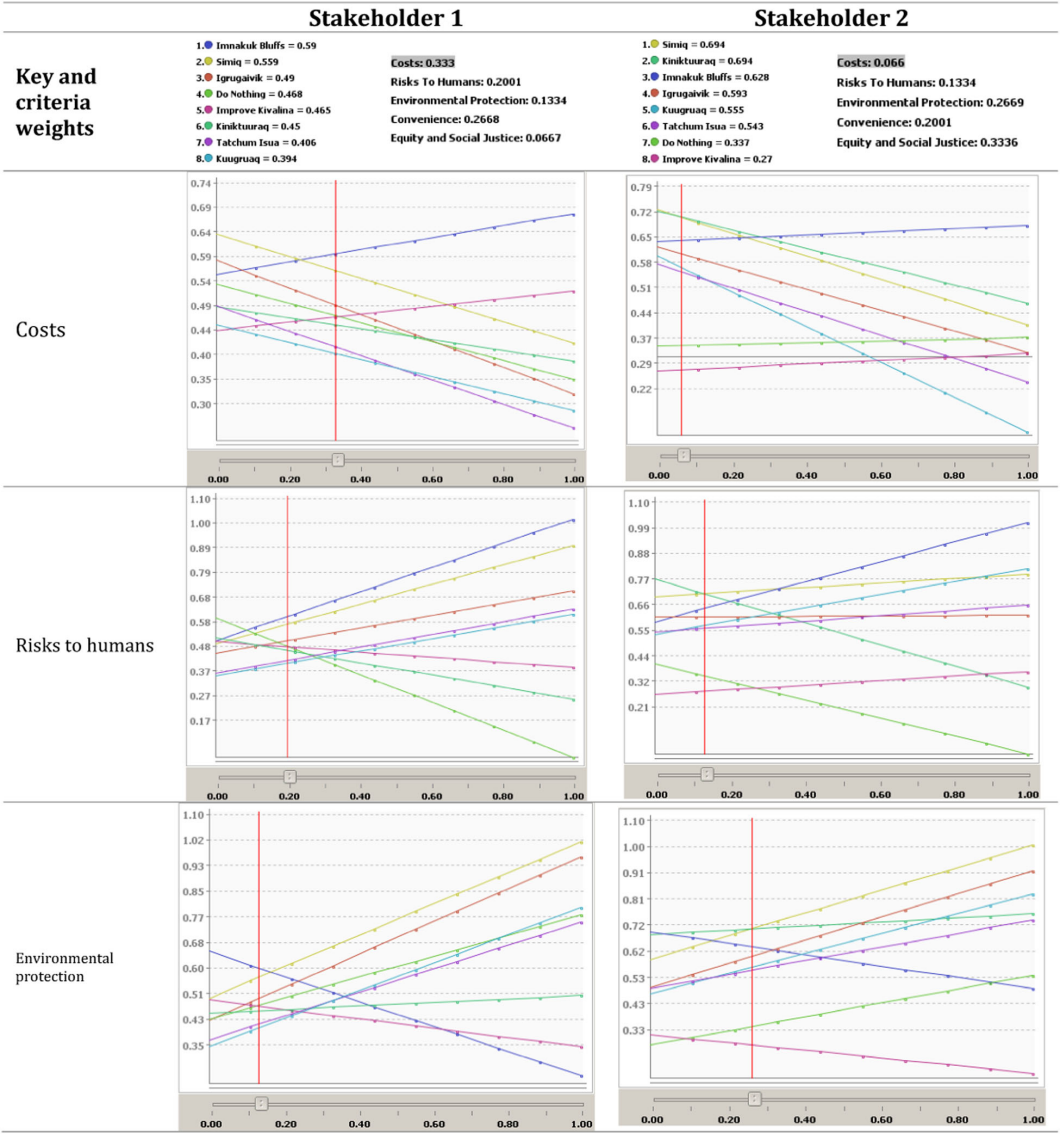

For example, if in the case of Stakeholder 1, the weight that the stakeholder places on “Costs” changes from its current weight of 0.33–0.20, Simiq becomes the preferred alternative as opposed to Imnakuk Bluffs. This reversal is pictured in the first row of Table 5, where 0.20 on the *x*‐axis would place the performance score of Simiq above Imnakuk Bluffs. Similarly, if Stakeholder 1 cared only slightly more for “Environmental Protection” to about 0.20 from its current weight of about 0.13, this would also make Simiq the preferred alternative. One can also see this logic work in reverse: as Stakeholder 1's weight on “Risks To Humans” increases, Imnakuk Bluffs only increases in its preferential status. When one looks at Stakeholder 2, one can see different preference changes. If Stakeholder 2 cared only slightly less for “Environmental Protection,” from about 0.27 to 0.20, the preferred alternative shifts from Simiq to Kiniktuuraq.

This is important information for decisionmakers, as it gives them a greater sense of which potential alternatives warrant greater scrutiny and allows them to visualize the point where a preferred alternative may no longer be preferred based on how much a stakeholder “cares” about that criterion. This can inform an agency's time and resource allocation efforts; in this notional scenario, if there is a way that USACE could bring down costs for the Simiq alternative, it could potentially be more favorable to Stakeholder 1. This could also inform an agency's efforts regarding which criteria to prioritize in mediation efforts. For example, if Stakeholder 1 was made to care about “Environmental Protection” only slightly more, both parties would then be in relative agreement regarding which alternative would be preferred. This demonstrates the usefulness of this tool in potentially reconciling stakeholder differences and arriving at the most agreeable alternative.

### Model limitations

There are several limitations to this model. First, as noted earlier, using stakeholders' ranks for criteria is a significant assumption. Ranks, which are then converted to weights, may not reflect the true difference in preference between one criterion and another.

As stated before, most of the criteria in this problem are inherently subjective. The use of a 1–10 scale for measuring criteria, such as “Political Feasibility” or “Fairness and Equity,” requires a level of honest, unbiased professional judgment to ensure that the model produces the outcomes that most accurately reflect the confluence of alternative performance and stakeholder input. In some coastal retreat situations, more objective metrics to measure these subjective criteria may be available, which can easily be incorporated into the framework presented in this paper.

Agency bias can also be an issue in scoring alternatives against criteria and assigning weights to specific stakeholders. There could be the conscious or unconscious temptation to score alternatives and elicit stakeholder input in such a way that the output of the model “favors” whatever outcome is the favorite of the agency. This potential exists in MCDA (Roy, 2005), but policymakers must hold themselves to the highest levels of scrutiny to account for and be aware of this potential.

Finally, and perhaps most importantly, the criteria outlined in this model may not be fully sufficient to cover the unique situations of *every* single locality that must evaluate a set of coastal retreat alternatives; in some situations, one or more new criteria may need to be introduced. While this may be the case, the intent of this model is to provide a broad, strategic‐level starting point for government agencies to begin framing, planning for, and evaluating coastal retreat policy—not to be the final say in every coastal retreat problem. In fact, the insights to be gleaned from a group of stakeholders in a community devoting thought to local issues that may be missed by this model is part of the reason this model was created. This model is not only meant to guide decision‐making, but also to provide a forcing function or anchor to help government agencies spur thought about community and stakeholder values, and ultimately make more informed decisions.

### Future work

While there is no doubt that climate change will profoundly affect those living on the coasts, there remains great uncertainty regarding the precise extent of its effects. As such, there is a temporal aspect of this problem that must be considered when deciding what communities will need to move and where. This consideration is addressed with the “Flexibility to Change and Adaptation” criterion, but in its current form, it will be up to the user to identify a timeframe to bound the model. Future improvements to this or a similar framework could incorporate DAPP to address the time‐based aspect of this problem more effectively (Lawrence et al., 2019).

On this note, this uncertainty creates the need for value of information analysis to gauge the sufficiency of present information, understand knowledge gaps, and determine whether certain decisions ought to be made at a given time (Wilson, 2015).

The framing of this model was partially informed by meetings with the Martha's Vineyard Commission in Massachusetts. While this community is beset by the effects of sea level rise like the others, they will examine this problem from a different perspective than those of other communities. Structured meetings with other localities, such as the Inupiat of Kivalina and Shishmaref, could potentially refine and improve this framework.

Similarly, since all localities which inform this model are in the United States, the current framework may be inadequate to fully capture the scope of managed retreat scenarios in localities outside the United States. The incorporation of international case studies could potentially address this shortcoming.

This model does not prescribe a methodology for how decisionmakers can identify coastal retreat alternatives. The utility of this model would largely be dependent on the alternatives, which it is evaluating, and a framework for the selection of alternatives could improve the usefulness of this tool.

Lastly, this model does not specify *how* agencies should identify stakeholders in coastal retreat scenarios, nor does it provide specific guidance on the elicitation of stakeholder preference. The former is an issue which is beyond the scope of this paper and could likely be a topic of future work. For the latter, as stated before, the decision to rank criteria on a scale of most to least important was made to simplify the process of working with stakeholders without well‐defined preferences, but policymakers may find that this, too, is insufficient. Future work could include how to use techniques such as fuzzy cognitive mapping for preference elicitation (Kosko, 1986). As with many limitations to this model, this requires the professional judgment of decisionmakers.

## CONCLUSION

Multicriteria decision analysis provides a structured way for stakeholders and decisionmakers to frame and interact with the problem of coastal retreat. It is a tool that, in addition to potentially providing the best course of action, helps those involved develop and elucidate preferences and common understanding. The implementation of an effective managed retreat policy will require not just the input and collaboration of communities and stakeholders with government agencies, but an evaluation mechanism through which organizations charged with executing retreat policy can balance competing demands. With the strategic multiobjective framework presented in this paper, relevant agencies can make more informed decisions regarding coastal retreat policy and accomplish this in a way which most closely reflects the values of all parties involved.

## CONFLICT OF INTEREST

The authors declare no conflicts of interest.

### Open Data Badge

This article has earned an Open Data Badge and Open Material Badge for making publicly available the digitally shareable data necessary to reproduce the reported results. The data and material are available at https://osf.io/a3v4x/. Learn more about the Open Practices badges from the Center for Open Science: https://osf.io/tvyxz/wiki.

## Data Availability

All data and calculation tools for this work are available for reproduction through OSF at the following link: https://osf.io/a3v4x/. This work utilizes the multicriteria decision analysis platform Decerns.
